# Development and Maintenance of Epidermal Stem Cells in Skin Adnexa

**DOI:** 10.3390/ijms21249736

**Published:** 2020-12-20

**Authors:** Jaroslav Mokry, Rishikaysh Pisal

**Affiliations:** Medical Faculty, Charles University, 500 03 Hradec Kralove, Czech Republic; rishikaysh2007@gmail.com

**Keywords:** stem cell, epidermal placode, skin adnexa, signalling, hair pigmentation, markers, keratins

## Abstract

The skin surface is modified by numerous appendages. These structures arise from epithelial stem cells (SCs) through the induction of epidermal placodes as a result of local signalling interplay with mesenchymal cells based on the Wnt–(Dkk4)–Eda–Shh cascade. Slight modifications of the cascade, with the participation of antagonistic signalling, decide whether multipotent epidermal SCs develop in interfollicular epidermis, scales, hair/feather follicles, nails or skin glands. This review describes the roles of epidermal SCs in the development of skin adnexa and interfollicular epidermis, as well as their maintenance. Each skin structure arises from distinct pools of epidermal SCs that are harboured in specific but different niches that control SC behaviour. Such relationships explain differences in marker and gene expression patterns between particular SC subsets. The activity of well-compartmentalized epidermal SCs is orchestrated with that of other skin cells not only along the hair cycle but also in the course of skin regeneration following injury. This review highlights several membrane markers, cytoplasmic proteins and transcription factors associated with epidermal SCs.

## 1. Epidermal Stem Cells as Units of Development

### 1.1. Development of the Epidermis and Placode Formation

**Figure 1:**
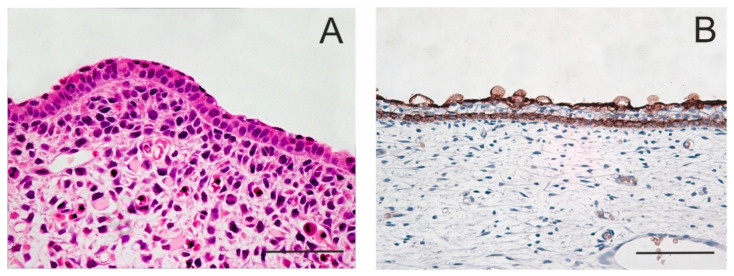
Periderm in embryonic epidermis. The periderm forms the outermost layer of embryonic skin that looks like a single superficial layer of flattened cells which functions as a permeability barrier. E12 mouse; staining with haematoxylin–eosin (**A**). After the appearance of an intermediate cell layer, the periderm becomes separated from the basal layer. Skin of a 14-week human embryo; anti-keratin 19 immunoperoxidase staining (**B**). Scale bar 50 μm.

The embryonic skin at very early stages of development is covered by a surface ectoderm that is a precursor to the epidermis and its multiple derivatives. As the embryo grows, these ectoderm cells undergo symmetric divisions which increase a pool of equal epithelial cells sharing the same characteristics. They express (cyto)keratins 8 (K8) and K18, which are the first intermediate filament proteins expressed in mouse development. An additional layer of flattened and well-adherent cells, the periderm (epitrichium), starts to form from embryonic day 9 (E9) in mice to protect the body surface exposed to the amniotic fluid. At E9.5, keratins K8 and K18 are replaced by K5 and K14, which mark epidermal commitment [1,2]. Since E10.5, Myc proteins drive epidermal cell competition, during which unfit cells (losers) that are prone to apoptosis are replaced by more stable progenitors (winners) that express engulfment machinery genes [3,4]. An increase in the number of epidermal cell layers requires another mode of cell division. Basal cells proliferate and form an intermediate cell layer under the periderm [1,5] (Figure 1); suprabasal cells divide and, from E13.5, start to express K1/K10. The development of skin appendages is associated with local cell divisions that give rise to epidermal thickenings. The production of Wnt signals in these progenitor cells is polarized: Wnt inhibitors are released apically, while Wnt ligands are delivered toward the underlying mesenchymal cells, which creates a sharp morphogen gradient that drives placode development [6]. Unequal daughter cells are generated by asymmetric divisions, leaving WNT^hi^ cells—which are assembled into the placode—anchored to the basal lamina and displacing WNT^lo^ cells to a suprabasal layer [7]. SHH produced by WNT^hi^ cells instructs the adjacent WNT^lo^ cells to expand by rapid symmetric cell divisions. Signalling from the early placode induces the underlying mesenchymal cells to upregulate cell cycle inhibitors that suppress proliferation, which is followed by cell adhesion promotion in the surrounding fibroblasts. FGF20 released from the placode (as a result of its local Wnt activation) initiates fate specification of mesenchymal precursors cells by triggering the expression of the transcription factors Twist2, Lef1 and Smad3 and of signalling factors that include Dkk1, Cyr61, and Fst [8]. FGF20 that is still produced by placode progenitors acts on Sox2^+^ embryonic fibroblasts, promoting their migration and aggregation in dermal condensate just under the placode. Dermal condensate cells are then characterized by the expression of transcription factors like Trps1, Tshz1, Foxd1, Prdm1 and Sox18 and of the signalling proteins Fgf10, Ltpb1 and Sema6a. The crosstalk between this mesenchymal condensate and the overlying epithelial stem cells (SCs) directs differential fates of the overlying placodes into diverse skin adnexa. The density of placodes in the developing skin is regulated by competition between Wnt ligands (acting as placode promoters) and secreted inhibitors. Within each placode, Wnt stimulates the expression of a diffusible Wnt inhibitor, Dickkopf 4 (Dkk4), that suppresses placode fate in the epidermis adjacent to a placode [9,10]. In distant places where Dickkopf concentration ceases, Wnt activation induces the formation of new placodes. Epithelial ectodysplasin (EDA) signalling not only participates in placode formation but also supports the production of Dkk4.

The skin placodes are highly versatile structures. Throughout evolution, placodal SCs of different species were subjected to various types of modulation so to form diverse appendages like scales, feather follicles, hair follicles (HFs) and skin glands. Although the mechanisms directing the activity of multipotent cells in the epidermal placode have ancient roots and involve a similar signalling Wnt–(Dkk4)–Eda–Shh cascade, yet substantial differences exist in the development of different skin appendages. In the course of evolution, a careful regulation of epidermal SCs activity allowed to establish desired properties that provide a species with certain advantage. The formation of HFs and feathers requires the suppression of bone morphogenetic protein (BMP) signalling, whereas the same signalling promotes sweat glands and scales determination [11,12,13]. In embryonic mammalian skin, the antagonistic interplay of two different signalling pathways can block the formation of one appendage type and specify another. For example, in primates a precise temporally regulated balance in BMP–SHH antagonism allowed the transformation of some epidermal buds into hair follicles prior sweat glands formation [13].

### 1.2. Development of Hair Follicles

**Figure 2:**
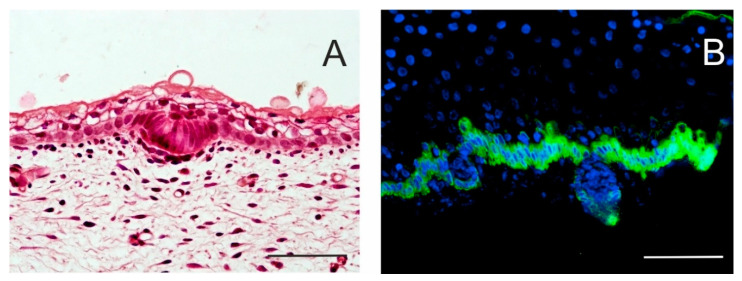
Initial stages of hair follicle (HF) formation. Local proliferation of epidermal stem cells results in epidermal thickening, characterizing an epidermal placode. Basal columnar cells communicate actively with mesenchymal cells that cluster under the basement membrane. Skin of a 14-week human embryo, haematoxylin–eosin staining (**A**). In a 17-week human embryo, the epidermis becomes thicker, and hair buds invaginate deep into the dermis to form hair pegs. Intense keratin 19 immunostaining is specific for the basal layer of the epidermis; some keratin 19-positive cells persist in hair pegs and periderm (**B**). Scale bar 50 μm.

In mice, the genesis of HFs begins around embryonic day 13 and occurs in well-orchestrated waves. At that time, the distribution of epidermal growth factor receptor and keratinocyte growth factor receptor (FGFR2-IIIb) in back skin epithelium is uniform but it is sharply diminished in E14.5 hair placodes. At that timepoint, Lgr6^+^ cells appear in the placodes. Meanwhile, mesenchymal cells underneath the hair placode cluster in dermal condensate, with a characteristic high expression of transcription factors including Hey1, Hes5, Glis2, FoxP1 and Alx4 and ligands such as Igfpb4, Inhba, and Rspo3, which are also specific for dermal papillae (DP) [8]. Dermal condensate cells produce the BMP inhibitor Noggin that stimulates the multiplication of the overlying WNT^hi^ cells within the hair placode [14]. Suprabasal WNT^lo^ cells, influenced by paracrine SHH signalling, express the HFSC master regulator SOX9; the progeny of these cells will later act as HFSCs. These cells rapidly undergo symmetrical divisions that lead to hair germ (HG) invagination [7] (Figure 2). The growing follicular hair bud engulfs the dermal condensate; a lower part of this hair peg contains LHX2^+^ cells [15]. Sox9^+^ and Lrig1^+^ progenitor cells are precursors of different HFSC subpopulations [16]. A growing hair germ develops in the bulbous peg; its upper two-thirds accommodate K17^+^ cells, which otherwise occur also in the basal layer of interfollicular epidermis. Sox9^+^ cells pile up in a bulge region, while Lrig1^+^ cells occupy HF upper part [16,17]. Otherwise, basal layers expressing K5, K14 and α6β4 integrin constitute the outer root sheath (ORS) that is continuous with matrix cells in contact with the DP, a clump aggregated from the underlying dermal condensate. DP signals the overlying hair germ/matrix cells to divide asymmetrically and produce multipotent progenitor cells, which express several transcription factors including Lef1 and Msx-2 and initiate cell differentiation leading to the formation of the inner root sheath and primitive hair shaft. The inner root sheath consists of several distinct cell layers that are concentrically arranged. No new HFs are formed postnatally. At birth, the HF is almost mature, and by P16 in mice the hair shaft reaches its full length.

### 1.3. Hair Pigmentation and Melanocyte SCs

Melanocyte SCs (McSCs) are not epidermis derivatives, as they are of neuroectodermal origin. By E8.5, the downregulation of Foxd3 leads to the activation of microphthalmia-associated transcription factor (MITF) that initiates the differentiation of neural crest SCs to the melanogenic cell lineage [18]. The precursors of the melanogenic cell lineage co-express MITF, PAX3, SOX10 and KIT. They are unpigmented but express the pigment enzyme dopachrome tautomerase (Dct). In the period between E10 and E12.5, these cells migrate toward endothelin-positive (Edn3/Ednrb2) target destinations; a chemotactic response is mediated by EdnR [19]. Around E13.5, these cells enter the epidermis from the underlying dermis. The receptor c-Kit enables melanoblasts to enter the HF bulge as bulge cells express stem cell factor (SCF) [20]. In HF, some melanin appears at the stage of hair peg formation. At birth, the melanoblasts in the interfollicular epidermis (IFE) are localized in basal layers; in HFs, they reside as McSCs in bulge, while the hair matrix contains the first melanocytes characterized by the expression of MITF, PAX3, DCT, Trp1, TYR and Kit. 

### 1.4. Development of Sebaceous Gland

The genesis of a sebaceous gland (SG) is closely associated with the development of HF to form the pilosebaceous unit. The SG cell lineage appears at the stage of bulbous peg and since then, SG are connected to the upper part of the follicle that contains Lrig1^+^ SCs [16]. Asymmetrically dividing Lrig1^+^ cells give rise to sebocytes. Sox9^+^ SCs, that act as precursors of Lrig1^+^ cells, have also the potential to give rise to SG cells. A subset of Lrig1^+^ cells also expresses the transcription factor Gata6 which regulates sebaceous lineage specification. Gata6^+^ cells appear in the HF infundibulum, junctional zone, upper SG and its duct portion [21,22]. Other SC populations, expressing LGR6, MTS24/PLET1 and K15 and occupying the isthmus, junctional zone and bulge, can participate in the generation of SG progeny [23,24,25]. Signalling mediated by the transcription factors TCF3/Lef1 as downstream mediators of Wnt/β-catenin signalling and hedgehog (Hh) signalling play a principal role in the regulation of cell multiplication and differentiation in the course of SG development [16]. 

### 1.5. Development of Interfollicular Epidermis

In the IFE, the stratified skin that develops between HFs, and the basal layers are continuous with the ORS of HFs. Asymmetric cell divisions in the IFE increase the number of cell layers and promote Notch-dependent differentiation in epidermal cells. BMPs stimulate the basal cell layer to express the transcription factor p63 that allows epidermal cells to proliferate and differentiate. p63 also triggers the expression of the ligand Jagged in the basal cell membrane, which activates Notch receptor in the membrane of overlying cells that cease to divide and start differentiating into keratinocytes [26]. The expression of p63 remains high during development, and its role is restricted towards the commitment of ectodermal cells to K5^+^K14^+^ stratified epithelia. p63 also allows K14 expression and the maintenance of epidermal SC renewal [27]. The proportion of suprabasal cells to cells in the basal layer is constant, allowing the skin barrier to remain functional [28]. Clearance following epidermal stratification is activated in detriment of terminally differentiated cells that downregulate ribosomal genes and undergo upward efflux, leaving the winner cells in the innermost layer as epidermal stem cells [4]. Interestingly, the orientation of progenitor cell division (and, therefore, clonal orientation) correlates well with the orientation of collagen fibres in the underlying dermis [28]. By P4 in mice, pigment cells disappear from the IFE and remain only in HFs. 

### 1.6. Development of Other Cutaneous Appendages

The development of eccrine sweat glands (SwGs) begins with the formation of epidermal placodes composed of K14^+^ progenitor cells [29]. In mice, pre-germs appear by E16.5, first in the proximal footpads, i.e., SwGs develop after HF specification [13]. The underlying mesenchymal tissue is only a thin sheath surrounding a nascent gland, in contrast with what observed for HFs, that require condensation into DPs [30,31]. The regional mesenchyme is a source of BMP5; epithelial bud cells increase the expression of Bmpr1a and Engrailed-1 [13]. SwG germs show high expression of Dkk4, while Wnt signalling is reduced [31]. Eda signalling is also involved in the regulation of SwG formation. Shh is activated downstream of Eda and participates in secretory coil formation [31]. 

**Figure 3:**
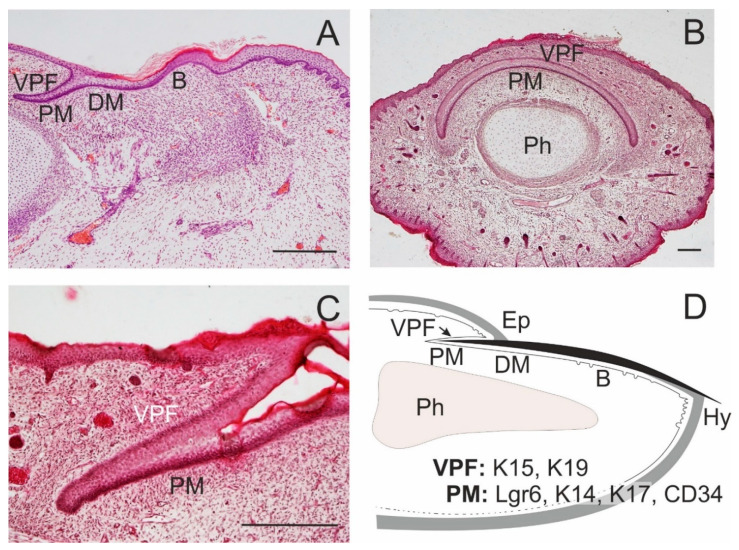
Formation of the nail. Nail proximal (PM) and distal matrix (DM) constitute a nail stem cell niche. In the dorsal side of a fingertip of a 14-week human embryo, a ventral proximal fold is in close association with PM; a nail bed (**B**) appears in continuation of the nail matrix (**A**). A transverse section of a distal tip of the little finger in a 17-week human embryo shows a curved course of PM, with overlying ventral proximal fold (VPF); distal phalanx (Ph; **B**); a detail of the association of PM and VPF in a longitudinal section (**C**). A schematic drawing of the arrangement of epithelial structures associated with an adult nail, with the distribution of some SC markers; eponychium (Ep), hyponychium (Hy; **D**). Scale bars 25 μm.

The development of other cutaneous appendages shares features and morphogenetic programs similar to those of HFs. Nail primordia (Figure 3A–C) arise from the development of epidermal placodes on the dorsal side of fingertips [32,33]. A well-tuned interplay with mesenchymal cells that involves Wnt signalling drives the proliferation of matrix cells under the proximal nailfold. The development of fingertips in the hands precedes the development of nails in the legs. Claws and hooves evolve as an adaptive diversification of nails.

## 2. Epidermal SCs as Units of Tissue Maintenance

### 2.1. Maintenance of the HF 

**Figure 4:**
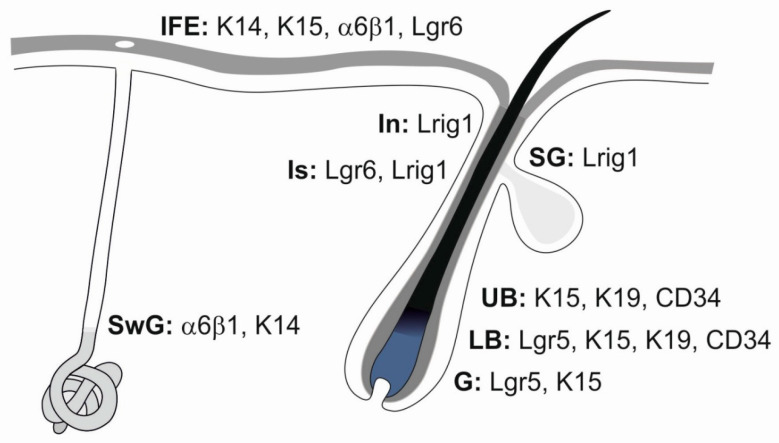
Heterogeneous stem cell populations residing in the epidermis. A schematic drawing represents interfollicular epidermis (IFE), sweat gland (SwG), sebaceous gland (SG), and characteristic morphologic parts of the hair follicle: infundibulum (In), isthmus (Is), upper bulge (UB), lower bulge (LB) and hair germ (G). Distinct stem cell populations can be characterized by specific markers. Modified from [41,43,44].

The HF is a miniorgan compartmentalized by several pools of epidermal SCs that inhabit distinct microniches (Figure 4). Lgr5^+^ and Gli1^+^ cells are also localized into a hair germ [34,35]. SCs in the bulge region express markers like CD34, K15, K19, Lrg5 and Sox9, K5, 14, TCF3/4, NFATc1, LHX2, Lgr6^lo^ and integrins α3β1 and α6β4 [36,37,38,39,40,41]. K15^+^CD34^+^ multipotent HFSCs occur in the lower portion of the anagen bulge; they comprise Lgr5^+^ or Lgr5^−^ subpopulations. The isthmus above the bulge is a site of Plet1^+^, Lrg6^+^ and Gli1^+^ SCs, and the junctional zone near SG opening contains cells expressing Lrig1 and Plet1. The infundibulum bears Sca1^+^ cells [42].

SCs in the mid-bulge of the HF express genes facilitating the formation of tendons and ligaments and produce nephronectin, which is deposited on the adjacent basal lamina [45,46]. This creates a specific niche that is recognised by smooth muscle myoblasts which form the arrector pili muscle. The overexpression of genes involved in the formation of tendons and ligaments is responsible for leaving this bulge area free of vascular and nerve supply, which contributes to the quiescence of the residential SCs. Laminin 332, as a component of the basal lamina, allows anchoring COL17A1^+^ SCs in a bulge; the anchorage is crucial for the maintenance of HFSCs [47]. The bulge region is also enriched with the proteoglycan decorin [48] that can bind TGF-β1 and interact with EGFR. The upper bulb SCs produce another extracellular matrix (ECM) molecule, EGFL6, that is released by upper bulge cells together with BDNF and is required for the sensitive innervation and the organisation of lanceolate mechanosensory complexes. Sensory nerves, on the other hand, deliver Shh signals that participate in the specific regulation of gene expression in the upper bulge [35]. Moreover, Shh–Gli signalling is helpful to hair-associated melanocytes [35].

The bulge contains long-term SCs that are kept in quiescence during a resting phase (telogen) by signals from BMP6 and FGF18 released by inner differentiated cells [49]. Signals from adjacent cells and DP including Wnt and BMP inhibitors induce Lgr5^+^ SCs to enter a new cell cycle. Growth-activating Wnt/β-catenin signalling is controlled in part by Kindlin-1 that interacts with cell membrane integrins. Expression of Runx1 by hair epithelial cells helps to reorganize blood capillaries adjacent to the hair germ in a pattern coordinated with the hair cycle [50]. 

In the next growth phase, i.e., anagen, new WNT^hi^ multipotent progenitors arise and express SHH, which stimulates bulge SCs to proliferate and form new ORS cells. A growing ORS elongates the basement membrane, increasing the distance from DP signalling, which allows bulge SCs to return to a dormant state. After ORS progenitor cells exhaust their cycling capacity, hairs stop growing, progenitor and matrix cells undergo programmed cell death, and HFs degenerate into the catagen phase. Some Lgr5^+^ SCs spared in the ORS constitute a new bulge and hair germ, which after remodelling and proper signalling, are able to enter a new hair cycle.

As hairs arise from modifications of ancient reptilian scale placodes similarly to feathers and scales of extant amniotes, all these appendages share some general structural features [51]. They contain clonogenic multipotent epidermal SCs harboured in a niche. These SCs can be visualized via bromodeoxyuridine label retention, as they are slow-cycling and share the expression of K15. More pieces of information are available about feather follicle stem cells that are characterized by the expression of the markers K15, K19, β1 integrin, CD49c (α3 integrin) and nestin [52,53]. Other properties related to HFSC behaviour include the ability to be moulded into different feather forms during development and to be activated with each feather regeneration cycle postnatally. 

### 2.2. Maintenance of McSCs

The regulation of McSCs activity depends on the crosstalk with neighbouring cells through signalling that involves WNT/β-catenin, KITL/KIT, EDNs/EDNRB, TGF-β/TGF-βR and Notch (reviewed in [54,55]). McSCs sharing the bulge HF niche remain in close contact with K15^+^CD34^+^ HFSCs. HFSCs express the transmembrane protein collagen type XVII that participates in the construction of hemidesmosomes and interacts with α6 integrin. Collagen type XVII alpha 1 chain (COL17A1) is important for the maintenance of HFSCs and for the stabilization of McSCs adhesion [53]. Loss of COL17A1 is associated with hemidesmosome fragility and stem cell delamination, followed by depletion of neighbouring pigment cells [56]. Telogen HFSCs secrete the Wnt inhibitors Sfrp1, DKK3 and Dab2 that contribute to the maintenance of McSCs quiescence. MSCs express TGF-β that downregulates PAX3 and MITF, which suppress melanogenesis. Notch1/2-RBP-Jk signalling also participates in McSCs quiescence. Notch receptor and its target gene *Hes1* are expressed in quiescent McSCs; HFSCs-derived Jagged (which acts as a Notch ligand) keeps McSCs quiescent [20]. Other signals contributing to McSC dormancy include BMPs released form subcutaneous adipocytes. Quiescent McSCs are activated to self-renew in early anagen by paracrine signals from HFSCs and PDGF secreted by subcutaneous adipocytes [20]. Edn1/2 and Wnt released by HFSCs control the regeneration and migration of McSCs; PDGF from adipocytes also promotes melanocyte differentiation. The produced melanocyte progenitor cells move to the hair matrix at the bottom of each HF to initiate hair shaft pigmentation [55,57]. Hair colour is balanced by the production of different pigments. The black pigment eumelanin is produced after α-MSH binds MC-1R [58], whereas genesis of the yellow pigment pheomelanin is induced by BMP-4 acting via Agouti signal protein (ASP) from DPs, which normally competes with α-MSH [59]. 

### 2.3. Maintenance of SG 

SG is attached to a HF at sites above the arrector pili muscle. Concerning cytokeratin expression, dividing SG cells express K5 and K14. Lrig1 and Lrg6 expression appears in the basal layer that is responsible for SG homeostasis. Gata6^lo^ cells coexpress Lrig1 and Lrg6; postnatally, these cells are localized in the junctional zone, the upper part of SGs and sebaceous duct [21,22]. SGSCs are not dependent on Lgr6^+^ SCs from adjacent compartments comprising HFs and IFE. Blimp1^+^ cells associated with a duct can act as SGSCs. However, Blimp1 positivity was also observed in differentiated sebocytes [60] and thus cannot be considered a universal marker of SGSCs. 

### 2.4. Maintenance of IFE 

The linear postnatal growth of the IFE compartment ends around P30, and then its rate is reduced. These changes are accompanied by an increased heterogeneity of basal epidermal cells, as confirmed by single-cell RNA sequencing indicating the appearance of an additional stem/progenitor cell population and the transition to adult homeostasis [28]. The populations of Lgr6^+^ SCs in each epidermis compartment comprising HF, SG and IFE seem to be independent. In PW3, IFE Lgr6^+^ cells account for 5% of basal cells, in PW8, their numbers increase four times and at the age of 5 months, they represent 22% of basal epidermal cells [41]. Basal cells of the epidermis express K5 and K14 (like the HF ORS); stratifying cell layers committed to terminal differentiation are immunopositive for K1 and K10. Basal epidermal cells characterized by high p63 expression and low p63 phosphorylation represent epidermal SCs (p63^hi^pp63^lo^). The initiation of keratinocyte differentiation in IFE is accompanied by an increase in p63 phosphorylation; thus, differentiated epidermal progenitor cells show high p63 expression and high p63 phosphorylation (p63^hi^pp63^hi^) and appear mostly in suprabasal layers [61]. Expression of collagen XVII in the Malpighian layer helps to coordinate IFE cell proliferation [62]. COL17A1^hi^ cells divide symmetrically and sustain a high clonogenic capacity [56]. Epidermal cells expressing Toll-like receptor 7 (TLR7) possess SC characteristics and reside in the interfollicular region of the epidermis. TLR7-expressing cells can reconstruct the interfollicular epidermis and maintain intact interfollicular epidermal structures in 3D organotypic culture and serial transplantation assays, respectively. Thus, TLR7 might prove to be a useful marker to identify epidermal SCs along with K15, K19 and β1 integrin [63].

### 2.5. Maintenance of Nails 

Epidermal SCs occur also in other specialized skin appendages that include the nails (Figure 3D). SCs expressing K15 and K19 were described in the ventral proximal nail fold [64]. Highly proliferative K14^+^ and K17^+^ SCs are localized to the proximal matrix; these SCs are endowed with a high regenerative and clonogenic potential [65]. Less abundant Gli1^+^ SCs also share the proximal nail matrix [66]. Lgr6^+^ SCs localized in the nail matrix produce cells of the nail plate and participate in fingertip regeneration [33]. Additional SC markers identified in nail matrix cells include CD29 and CD34 [67].

## 3. Epidermal SC Markers 

Specific populations of epidermal stem cells are usually identified and isolated with the help of several markers whose distribution in the skin is schematically represented in Figure 4 and Figure 3D. As a high affinity receptor for R-spondins 1-3, leucine-rich repeat-containing G protein-coupled receptor 6 (Lgr6) modulates Wnt canonical signalling. Receptor stimulation upregulates the expression of several genes associated with Wnt signalling, including *Wnt6, Fzd1, Soc4* and *TCF712*, downregulates *Sostdc1* and *Htra1* involved in the negative regulation of BMP pathway and stimulates SC proliferation [68]. Lgr6^+^ epidermal cells renew the epidermal compartments they occupy [41]. Lgr6^+^ SCs located in the central isthmus are considered to be the most primitive epidermal SCs; postnatally, they renew IFE, sebocytes and cells of the HF isthmus. These cells can exit their local microenvironment, generate Lgr5^+^ SCs and contribute to the formation of the hair germ [69]. In the lower HF isthmus, LGR6^+^ cells co-express Gli1. Expression of Lgr6 was found to be related to the innervation of epidermal structures [70], and Lgr6^+^ cells also revealed the co-expression of several genes involved in axon guidance, like *Alcam*, *Sema3e*, *Ntf3* and *Nrp*. 

Lgr5 is considered a typical marker of several epithelial SCs including those in the mammary gland, kidney and stomach [71,72,73]. In the skin, Lgr5^+^ SCs reside in the lower bulge region and HG and are multipotent, capable of producing cells of HF, SG and IFE. These cells are characterized by active Hh signalling and the transcription of components of the Wnt cascade. The interaction of Lgr5 receptor with R-spondin inactivates membrane E3 ligase RNF43/ZNF3 ZNRF3 and enhances Wnt activity by preventing the ubiquitination and degradation of Wnt receptors [74]. The number of Lgr5^+^ SCs diminishes after deletion of *Lgr4*, which also promotes the activation of HFSCs [75]. 

The transmembrane protein LRIG1 (leucine-rich repeats and immunoglobin-like domains 1) was recognized as another marker of adult epidermal SCs [17,76]. LRIG1 mediates the ubiquitination and degradation of activated EGFR/ERBB and negatively regulates their signalling [77,78]. Lrig1^+^ cells are highly proliferative and maintain the upper pilosebaceous unit as an independent compartment, as they do not contribute to HF or IFE, which are maintained by distinct stem cell populations [76].

The expression of cytokeratins is closely related to epidermis development, and distinct pairs of keratins can be examined for the characterization of certain developmental stages and cell lineages (Table 1). Keratins associated with epidermal SCs include, above all, K15 and K19. Keratin 15 is mainly expressed by slow-cycling HFSCs occupying the bulge and (secondary) hair germ; these SCs have a large proliferative potential, and their progeny contributes to all epithelial cell lineages constituting the HF. These SCs co-express CD29 and CD34 (reviewed in [79,80]). Keratin 19 appears in the basal cell layer of the epidermis; its level in the developing epidermis is high and diminishes postnatally with skin maturation (Figure 1B and Figure 2B). Keratin 19 is also considered another marker of mouse bulge SCs. In humans, K19^+^ SCs appear at a later stage than SCs expressing K15, as a distinct cell population, predominate in the lower reservoir of anagen HF and likely represent a second SC reservoir. K19^+^ SCs are clonogenic in vitro, co-express α3β1 integrin and rarely co-express the proliferation marker Ki-67. Subpopulations with higher levels of K15 and CD200 are specific to higher reservoirs of HFs [79,81].

CD34 is a traditional SC marker. In the epidermis, CD34^+^ SCs occupy the hair bulge and represent HF label-retaining cells. Integrin α6^+^CD34^+^ SCs give rise to epithelial cells distributed in all layers of the epidermis. The population of α6^hi^ keratinocytes is enriched in small epidermal cells with high proliferative and clonogenic potential [84]. In human epidermal cells, strong expression of CD34 is observed in those cells occupying mainly the lower HF reservoir; CD34^−^ bulge SCs reveal low clonogenicity in a dish. A similar distribution was also described for CD271 (LNGFR or p75NTR) and CD 29 (β1 integrin) [79,85]. Apart from being a cell surface marker, β1 integrin is critically important for the expansion of epidermal SCs to maintain epidermal homeostasis [86]. Other integrins that have been used for the isolation of epidermal SCs include α2, α4 and β4 (reviewed in [87]). Loss of epidermal cell attachment to the ECM associated with altered integrin signalling induces a type of programmed cell death called anoikis. The protein survivin that accumulates in SCs of the basal epidermis layer participates in the regulation of cell division and suppresses the caspase 9-activated apoptotic pathway in association with XIAP, providing protection to SCs both in vitro and in vivo. The levels of this anti-apoptotic protein in SCs are related to those of β1 integrin, because blocking of this integrin thoroughly abrogates survivin expression in epidermal SCs [88].

Side-population (SP) cells enriched in stem cells efflux the fluorochrome Hoechst 33342 due to the expression of the membrane transporter BCRP1/ABCG2. Cells with this positivity were identified in the upper isthmus of mouse HFs [89]. SP HF cells are CD34^−^; they represent a heterogeneous cell population that comprises both α6^lo^ and α6^hi^ epidermal cells but differ from cells that are Lrig1^+^ and Lgr6^+^. Newborn and adult IFE also contains ABCG2^+^ cells [90]. The transporter belongs to a multidrug resistance protein family and provides the cells resistance against xenobiotic substances. The capacity to efflux the Hoechst dye is specific to 1.8% of HF cells. In addition, 85% of SP cells co-express the marker Plet-1 (placenta-expressed transcript-1) that is recognized by the mouse thymic stroma 24 monoclonal (MTS24) antibody [89].

Epidermal stem cells can be also characterized by the expression of specific transcription factors including Sox9, Gli1, Lef1, Lhx2 and others [35,91,92]. Often, varying levels of their expression or modification can help to distinguish SCs from progenitor cells. This is also the case for p63, which is highly expressed in epidermal SCs that show low level of p63 phosphorylation [61].

## Figures and Tables

**Table 1:** Epidermal keratins.

Keratin Type	Partner Keratin	Occurrence in Epidermis
K1	K10	differentiated keratinocytes in suprabasal layers [1,82]
K2	K10	terminally differentiated keratinocytes [1,82]
K5	K14	proliferative keratinocytes in the basal layer; myoepithelial cells [1,83]
K8	K18	earliest progenitors lost with skin maturation [83]
K9	K1	palmar and plantar epidermis [83]
K10	K1	differentiated keratinocytes in suprabasal layers [1]
K14	K5	proliferative keratinocytes in the basal layer [1,83]
K15	K5	HF SCs, restricted to basal cells of stratified epithelia [37,83]
K16	K6	hyperproliferative/migratory keratinocytes inducible in “activated” epidermis [82,83]
K17	K6	basal layer; inducible in “activated” epidermis; suprabasal layers in HF; contractile epithelium; myoepithelial cells [82,83]
K18	K8	earliest progenitors lost with skin maturation; IFE Merkel cells * [81,83]
K19	K7 **	SCs in HF bulge, basal layer of stratified epithelia; IFE Merkel cells *; eccrine sweat gland secretory cells *** [81,83]

Notes: * K18^+^ IFE Merkel cells coexpress K19 or K20. ** K7 in simple epithelia. *** Glandular secretory cells coexpress K8/18.
